# Human papillomavirus vaccine uptake in ethnically diverse women living with systemic lupus erythematosus

**DOI:** 10.1177/09612033251390599

**Published:** 2025-10-25

**Authors:** Sebastian Bruera, Yinan Huang, Savannah Bowman, Maria E. Suarez-Almazor, Grace H. Lo, Maria A. Lopez-Olivo, Elizabeth Chiao, Jennifer R Kramer, Frederick A. Pereira, Sandeep K. Agarwal

**Affiliations:** 1 Section of Immunology, Allergy, and Rheumatology, 3989 Baylor College of Medicine, Houston, TX, USA; 2 Department of Pharmacy Administration, 21693 University of Mississippi, Oxford, MS, USA; 3 Department of Health Services Research, 4002 The University of Texas MD Anderson Cancer Center, Houston, TX, USA; 4 Department of Epidemiology, 4002 The University of Texas MD Anderson Cancer Center, Houston, TX, USA; 5 Section of Health Services Research, 3989 Baylor College of Medicine, Houston, TX, USA; 6 Huffington Center on Aging, 3989 Baylor College of Medicine, Houston, TX, USA

**Keywords:** Systemic lupus erythematosus, anti-DNA antibodies, subacute lupus erythematosus

## Abstract

**Background:**

Women with systemic lupus erythematosus (SLE) are at an increased risk of infection from the human papillomavirus (HPV) and subsequently HPV-mediated malignancies and genital warts. The HPV vaccine is a highly effective intervention in preventing HPV infection and is recommended in SLE patients. We determined HPV vaccination rates and factors associated with decreased vaccination uptake in women living with SLE.

**Methods:**

We conducted a cross-sectional study in which we enrolled women with SLE (aged 21–45) for whom the HPV vaccine is recommended for by the US Food and Drug Administration (FDA). The primary outcome was self-reported HPV vaccination as recommended by the Advisory Committee on Immunization Practices (ACIP). We collected demographics, clinical characteristics, knowledge on HPV and vaccines, and items for constructs of the Health Belief Model (HBM) and determined associations between these covariates and HPV vaccination status.

**Results:**

We enrolled 75 women with SLE. Median age was 33 (IQR 27–40) and 20 (27%) had received HPV vaccination. Older women and Spanish-speaking patients were less likely to have received the HPV vaccine. When examining HBM constructs an increase in ‘perceived barriers’ (e.g. not knowing where to get the vaccine) was associated with no vaccination (r = −0.41, *p* < 0.01). Increased report of ‘cues to action’ (e.g. My doctors told me lupus increases risk for cervical cancer) was associated with increased HPV vaccination (r = 0.30, *p* < 0.01). After multivariable adjustment of significant covariates, age remained at significantly decreased odds for HPV vaccination (OR 0.82, 95% CI 0.73–0.93).

**Conclusion:**

We found low HPV vaccine uptake among racial and ethnically diverse women with SLE. Older age, Spanish language, increased perceived barriers, and increased cues to action were significantly correlated with HPV vaccination. This data highlights potential strategies for providers to use to improve HPV vaccination in this patient population.

## Introduction

Women with systemic lupus erythematosus (SLE) are at an increased risk of infection from human papillomavirus (HPV) and HPV-mediated malignancies and genital warts.^1–4^ Cohort data has shown that patients with SLE may have a two-fold increased risk of developing cervical cancer compared to general controls.^3^ More concerningly, a recent study has shown that patients with SLE have a three-fold increased risk of death from gynecologic cancers.^5^ The HPV vaccine is a highly effective strategy to prevent infection, and subsequently, these complications, and has been shown to be safe to administer in women with SLE.^6,7^

The Advisory Committee on Immunization Practices (ACIP) recommends routine vaccination at ages 12–26. However, these guidelines also recommend that patients who are at higher risk may consider receiving the vaccine through 45 years of age.^8^ Currently, there is a paucity of knowledge about HPV vaccination rates in women living with SLE, especially in racially and ethnically diverse patient populations. However, some studies have reported vaccine rates to be alarmingly low and consistently below 20%.^9–11^

Furthermore, few studies have assessed the knowledge, attitudes, and beliefs of women with SLE towards HPV vaccination, and to our knowledge no study has assessed this using the Health Beliefs Model (HBM), a conceptual framework frequently used to study determinants of engagement in preventative care.^12^ It is important to understand whether patients living with SLE are receiving HPV vaccinations and understanding potential barriers and attitudes to improve utilization of this intervention. The objectives of this study were to: (1) determine HPV vaccination rates in a racially and ethnically diverse population of women with SLE aged 21–45; (2) explore the knowledge regarding HPV vaccine that women with SLE have, and (3) Use the Health Beliefs Model (HBM) to determine their knowledge, attitudes, and beliefs towards HPV vaccination.

## Method

### Design

This study was granted approval by the Baylor College of Medicine and Harris Health Institutional Review Boards. All patients received informed written consent.

This study analyzed a subset of data from a previous cross-sectional study in which we explored cervical cancer screening rates in women with SLE aged 21–65.^13^ We administered a separate questionnaire pertaining to HPV vaccination to patients aged 21–45, as those older than 45 have not been recommended to receive the vaccine by ACIP best practice guidelines. We did not include patients under the age of 21 as cervical cancer screening guidelines are less clear for this patient population.

### Setting

The study was conducted at two clinical sites in Harris County, Texas. One site is the Harris Health outpatient rheumatology clinic, in which 54% of patients do not have public or private insurance and rely on a county publicly issued insurance called the “gold card”. In order to qualify for the gold card, the patients need to provide proof that the household income does not exceed the 150% of the federal poverty level.^14^ The second site is the rheumatology clinic from the Baylor College of Medicine Faculty Group Practice, which accepts public (including Medicaid) and private insurance.

### Population

We enrolled women with SLE (using the 2019 American College of Rheumatology or European League Against Rheumatism criteria) with no prior history of a hysterectomy or cervical cancer from July 2021 to December 2022.^15^ Patients were enrolled consecutively with convenience sampling after their regularly scheduled rheumatology clinic visits from July 2021 to December 2022.

### Data collection

Patients provided written consent, and then completed a survey which included demographics as well as knowledge, attitudes, and beliefs about HPV vaccination. The survey was provided in English or Spanish, according to patient choice. The Spanish version was generated using back-translation, in which one bilingual individual translated the English version to Spanish, and a second bilingual individual translated the Spanish version back to English.^16^

### Outcomes and covariates

The primary outcome of this study was HPV vaccination status according to recommendations from ACIP, which provides advice and guidance to the Center of Disease Control (CDC).^17^ These guidelines recommend that the HPV vaccination be given to all patients aged 9–26 years old and strong consideration of adults aged 27–45 that are immunocompromised. We considered that women had vaccination if they self-reported previous vaccination. As many women may have been children when they received the HPV vaccine and unaware, we asked that they contact their parents or kinship to inquire if they had received the vaccine. Possible answers about HPV vaccination status were yes, no, or unsure. In our statistical analysis unsure were treated as “No” as clinically both these patient populations may benefit from receiving the HPV vaccine. The methodology of self-reported vaccination was chosen as there are no widely accepted use of vaccination titers to determine HPV vaccine status at the time this study was conducted.

### Covariates

Demographics collected included age, education, income, insurance status, race, and ethnicity. We also collected the SLE Disease Activity Index (SLEDAI), the Systemic Lupus International Collaboration Clinics Damage Index, and drug therapy.^13^

### Health beliefs model and knowledge questionnaire

The Heath Belief Model (HBM) is a conceptual framework that has been used to explore the attitudes and beliefs of people towards preventive health care.^12^ The HBM has six paradigms to identify potential reasons that patients may not receive preventative health care and also potentially provide strategies to improve utilization of preventative care. These constructs include: (1) perceived susceptibility, (2) perceived severity, (3) perceived benefits, (4) perceived barriers, (5) cues to action, and (6) self-efficacy. Perceived susceptibility refers to an individual’s assessments of their risk towards developing the underlying disease, which in this study is HPV infection. Perceived severity refers to potential consequences after developing the disease (ex: I will get cancer if I am infected with HPV). Perceived benefits assesses patients’ understandings of benefits of the utilization of preventative healthcare (ex: The HPV vaccine will prevent HPV infections). Perceived barriers include any potential obstacles that may prevent patients from otherwise obtaining the preventative care utilization (ex: The HPV vaccine is too expensive). Cues to action reflect possible external factors that may improve vaccination efforts (ex: My doctors told me lupus increases my risk of cancer). Finally, self-efficacy refers to personal beliefs/factors that may improve vaccination efforts (ex: I see a gynecologist yearly). A systematic review has shown that utilizing the HBM to develop strategies to improve adherence has moderate to large effects (39%) in improving utilization of preventative care services.^18^ Respondents answer each item based on a Likert scale (1, - strongly disagree, 2-disagree, 3–neutral, 4–agree, 5–strongly agree).

As the HBM does not contain paradigms for patients’ knowledge towards cervical cancer screening and HPV vaccination, we developed a knowledge questionnaire for all patients (age 21–64). As no studies had previously conducted the HBM model in patients living with SLE, we reviewed previous published studies that have published both knowledge questionnaire and HBM instruments in cervical cancer screening and HPV vaccination in general populations to develop our own instruments.^19–25^ After administering 60 surveys, we estimated internal consistency with Cronbach’s Alpha for each domain of the HBM in which internal consistency of all items was robust except for self-efficacy.^13^ A systematic review showed that 78% of studies that using the HBM for implementation strategies had significant improvements in adherence.^18^

### Statistical analysis

We used descriptive statistics to summarize demographics of the study participants for the HPV vaccination. For the knowledge questionnaire, we used the proportion of correct answers.

For the HBM constructs, we used descriptive statistics to estimate the median, the mean and standard deviation for the Likert scale scores for each item. We then estimated the score for each construct by calculating the mean of the individual items under each construct. We generated Pearson correlation coefficients to evaluate the association between the constructs and individual items and HPV vaccination.

We used chi-square tests to examine differences between proportions, and Pearson correlation coefficients to estimate the association of HPV vaccination status with the covariates of interest. Finally, we conducted a multivariable logistic regression model for covariates that were significantly associated with HPV vaccination including Age (continuous variable), language (bivariate), perceived barriers (continuous), and cues to action (continuous).

## Results

### Demographics

From our initial cohort of women with SLE (*n* = 130), 75 were aged between 21 and 45, and therefore had a recommendation to receive the HPV vaccine based on ACIP guidelines. Baseline demographics are shown in Table 1. The median age was 33 (IQR 27–40). The patient population was racially and ethnically diverse (56% White, 20% Black, 44% Hispanic), including English speakers (60%), Spanish speakers (21%), and bilingual women (19%). Most patients had publicly provided insurance (such as Medicaid, Medicare) or county-provided insurance (63%).

**Table 1.** Demographics with vaccination rates (for HPV vaccine population only).

	Number (%)^a^	Self-reported “yes” HPV vaccination, *N* (%)^b^	No/Unsure vaccination status^b^	P^c^
Total	75 (100)	20 (27)	55 (73)	
Age (median, IQR)	33 (27-40)	28 (23-30)		**0.0003**
Age (21–26)	17 (23)	10 (59)	7 (41)	
Age (27–45)	58 (77)	10 (17)	48 (83)	
Race				
White	42 (56)	10 (24)	32 (76)	0.74
Black	20 (20)	5 (25)	15 (75)	
Asian	5 (7)	2 (40)	3 (60)	
American Indian or Alaska native	7 (9)	3 (43)	4 (57)	
Native Hawaiian	1 (1)	0	1 (100)	
Ethnicity				
Hispanic	33 (44)	10 (30)	20 (70)	0.87
Language				
English	45 (60)	17 (38)	28 (62)	**0.03**
Spanish	16 (21)	1 (6)	15 (94)	
Bilingual	14 (19)	2 (14)	12 (86)	
Insurance				
Private insurance	28 (37)	10 (36)	18 (64)	0.1
Public insurance	17 (23)	6 (35)	11 (65)	
Others	30 (40)	4 (13)	26 (87)	
Marital status				
Married	24 (32)	3 (13)	21 (87)	0.15
Single or never married	35 (47)	11 (31)	24 (69)	
Widowed, separated or divorced	16 (21)	6 (38)	10 (62)	
Disease activity				
Low (SLEDAI <6)	56 (75)	15 (27)	41 (73)	0.97
High (SLEDAI ≥6)	19 (25)	5 (25)	14 (75)	
Education				
Less than high school or high school or trade or technical school	67 (49)	8 (12)	59 (88)	0.46
Some college or bachelor	51 (38)	10 (20)	41 (80)	
Advanced degree or higher	18 (13)	2 (11)	16 (89)	

^a^Percentage in column is representative of total percentage of sample size (ex: Ages 21–26 were 17 of the 75 total patients).

^b^Percentage in column is representative of total percentage of that row (ex: Of the 17 patients aged 21–26, 10 received the vaccine).

^c^p for comparison between vaccinated and not vaccinated.

### HPV vaccination rates

Table 1 shows the HPV Vaccination rates. A total of 20 (27%) of women self-reported having received the HPV vaccine, 38 (52%) had said no, and 15 (20%) were unsure. Comparing those who reported receiving the vaccine and those who had not, no statistically significant differences were observed based on race, ethnicity, insurance, disease activity as determined by SLEDAI, or education. Younger patients and English-speaking patients were more likely to have received the HPV vaccine (*p* < 0.05). We found that 60% of patients aged 21–26 had received the HPV vaccine, whereas 17% of patients aged 27–45 had not.

### Knowledge questionnaire

Table 2 shows the results of the knowledge questionnaire. We administered this questionnaire to all patients (*n* = 130). The full questions with answer choices are shown in Table 2. Percentage of correct answers varied from 24% to 79%. Most patients were aware that HPV is the virus associated with cervical cancer and detection is via pap smear (94 (72%), 103 (79%)). The lowest scores in the knowledge questionnaire were for non-cancerous manifestations of HPV, and risk factors for HPV (31 (24%), 34 (26%)).

**Table 2.** Knowledge questionnaire (for all patients).

Questions	Correct percentile
Q: Cervical cancer is associated with which of the following viruses?	72%
A: 1. Epstein-barr virus (also known as “mono”) | 2. Herpes | 3. Human papillomavirus (HPV) | 4. HIV | 5. Don’t know	
Q: The virus associated with cervical cancer is transmitted by	
A: 1. Sexual intercourse | 2. Maternal-fetal transmission (when the mom passes it along to the baby) | 3. Blood transfusions (receiving somebody else’s blood) | 4. Surfaces (such as using a computer a stranger just used) | 5. Don’t know	54%
Q: Cervical cancer can be diagnosed by	
A: 1. X-rays | 2. Pap smear tests | 3. Blood tests | 4. Urine tests | 5. Don’t know	79%
Q: Who can be affected by the human papillomavirus (HPV)?	
A: 1. Males only | 2. Females only | 3. Both males and females | 4. Don’t know	67%
Q: The human papillomavirus (HPV) can be detected by	
A: 1. Pap smear | 2. Urine tests | 3. Blood tests | 4. Don’t know	60%
Q: Human papillomavirus (HPV) can cause	
A: 1. Vaginal discharge | 2. Genital warts | 3. Itching | 4. Burning with urination | 5. Don’t know	24%
Q: Which of the following increases my risk of HPV?	
A: 1. Lupus | 2. Sexual intercourse before age 18 | 3. Cigarettes | 4. Multiple sex partners | 5. All of the above | 6. I don’t know	26%
Q: Cervical cancer is preventable. (true or false)	76%

### Health beliefs model

The results of the HBM questionnaire to explore attitudes and beliefs towards HPV vaccination are shown in Table 3. Domains included were perceived susceptibility, perceived severity, perceived barriers, perceived benefits, self-efficacy, and cues to action.

**Table 3.** Correlation of health beliefs model constructs and individual items with self-report of prior HPV vaccination.

Domains (likert scale, 1, - strongly disagree, 2- disagree, 3 – neutral, 4 – agree, 5 – strongly agree)	Mean	SD	Correlation with vaccination (r)	P
Perceived susceptibility (higher scores indicate increased perceived susceptibility)	3.19	0.96	−0.06	0.61
I feel I will get HPV some time during my life	3.57	1.22	−0.09	0.45
I worry about getting HPV.	3.25	1.43	−0.17	0.15
Compared with other women, my chances of getting HPV are high	3.23	1.15	0.07	0.57
Lupus increases my chances of getting HPV.	2.72	1.07	0.04	0.74
Perceived severity (higher scores indicate increased perceived severity)	2.56	0.67	−0.02	0.88
If I had HPV, I would likely get genital warts	2.93	0.96	−0.04	0.72
If I had HPV, I would likely get cervical cancer	2.88	0.97	0.05	0.67
The thought of HPV scares me	2.12	1.15	−0.04	0.72
If I had HPV my whole life would change	2.29	1.19	−0.003	0.97
Perceived barriers (higher scores indicate increased perceived barriers)	3.66	0.81	**−0.41**	**0.0003**
I am afraid of the HPV vaccine because of possible side effects	3.37	1.17	**−0.27**	**0.02**
I do not think the HPV vaccine will help me	3.65	1.08	**−0.28**	**0.02**
I do not know how to get the HPV vaccine	3.45	1.33	**−0.41**	**0.0003**
I worry about the costs of the HPV vaccine	3.74	1.18	**−0.26**	**0.02**
I don’t have the time to get the HPV vaccine	4.07	0.99	−0.17	0.14
Perceived benefits (higher scores indicated increased perceived benefits)	1.90	0.68	−0.01	0.91
The HPV vaccine lowers the risk of getting HPV.	1.97	0.91	−0.15	0.20
The HPV vaccine lowers the risk of getting cervical cancer	2.07	0.93	−0.15	0.19
The HPV vaccine will lowers the risk of getting genital warts	2.42	1.02	−0.04	0.71
Self-efficacy (higher scores indicated increased self-efficacy)	1.97	0.78	0.02	0.88
I can go to hospitals or clinics and get the HPV vaccine	1.73	0.73	0.03	0.81
I see a gynecologist yearly	2.21	1.31	0.006	0.96
Cues to action (higher scores indicate increased cures to action)	1.98	0.38	**0.30**	**0.0083**
My doctors have told me I am high risk for HPV.	1.97	0.61	0.22	0.06
My doctors told me lupus increases my risk for cervical cancer	1.92	0.66	0.11	0.34
My doctors told me lupus increases my risk for HPV.	1.91	0.70	**0.27**	**0.02**
Having known someone with HPV makes me want to go get the HPV vaccine	2.01	0.67	0.19	0.10
I have a relative or close friend with HPV.	2.08	0.51	0.09	0.42

Of these domains, the *perceived barriers* and *cues to action* domains of the HBM were significantly correlated with decreased vaccination administration. Regarding the *perceived barriers* domain, the average response was 3.66 (SD = 0.81) which strongly correlated with HPV vaccine administration (r = −0.41, *p* = 0.0003). Within this domains, the most strongly correlated items included lacking knowledge on how to obtain the vaccine, potential costs about the vaccine, and beliefs that the vaccine would not be effective. In contrast, concern for side effects had only a minor correlation with administration of the vaccine and having enough time was not correlated with vaccine administration.

With regards to *cues to action*, respondents had low scores in the disagree range (mean = 1.98, SD = 0.38). Notably the scores in the *cues to action* domain correlated with HPV vaccine administration (r = 0.30, *p* = 0.009), suggesting that most patients had not received cues to action. Interestingly, one item in this domain was significantly associated with having received vaccination, “My doctors told me lupus increases my risk for HPV” (mean = 1.91, SD = 0.70, r = 0.21, *p* = 0.02), indicating that patients were more likely to receive HPV vaccination when doctors counseled patients with SLE about their increased risk of HPV.

With regards to *perceived susceptibility*, respondents on average had a neutral response *(*mean = 3.19, SD = 0.96, r = −0.06, *p* = 0.61). Regarding *perceived severity*, respondents also had a neutral response *(*mean = 2.56, SD = 0.67, r = −0.02, *p* = 0.88). *Perceived benefits* (mean = 1.90, SD = 0.68, r = −0.01, *p* = 0.91) and *self-efficacy* (mean = 1.97, SD = 0.78, r = 0.02, *p* = 0.88) were not significantly associated with prior vaccination. There were no individual items within these HMB that were significantly correlated with decreased vaccination.

### Multivariable logistic regression model

Finally, we performed a multivariable logistic regression model (Table 4) using the covariates identified above that were significantly associated with HPV vaccination. After adjustment for age, language, perceived barriers, and cues to action, older age remained significant, with lower odds of receiving the HPV vaccine (OR 0.82, 95% CI 0.73–0.93).

**Table 4.** Multivariable logistic regression model.

Effect	Odds ratio	95% wald confidence limits
Age	0.82	0.73–0.93
Language (Spanish vs English)	0.38	0.04–3.93
Language (bilingual vs English)	0.23	0.04–1.54
Perceived barriers	1.77	0.73–4.31
Cues to action	0.15	0.01–1.93

## Discussion

Given the increased risk of HPV infection in women SLE and the potential importance of HPV vaccine in preventing HPV infections in this high-risk population, this study sought to determine the percentage of patients that have self-reportedly received the HPV vaccination according to ACIP best practices and factors that are associated with decreased adherence these guidelines. This is particularly important as immunosuppression, which many patients living with SLE require chronically, is associated with HPV persistence and abnormal pap smears.^26^ We found that in a racially and ethnically diverse patient population that relies on mostly public insurance only 27% of patients reporting receiving the HPV vaccine. Univariate analyses demonstrated that older age and Spanish-speaking patients were less likely to have received the HPV vaccine. Finally, using the HBM model, we were able to identify that perceived barriers to HPV vaccination and cue to action were associated with prior HPV vaccination administration.

Our study is similar to previous studies in patients with SLE in which low rates of HPV vaccination have been observed.^10,11^ A previous cross-sectional study that enrolled African American women with SLE found that only 17% of patients had received the HPV vaccine.^10^ A second retrospective study using data from electronic medical records found that only 16% of patients had received the HPV vaccine.^11^ However, low rates of HPV vaccination have been seen in various patient populations including transplant recipients and the general population. Transplant recipients are on chronic immunosuppression and also follow the same guidelines as patients with SLE in which those up to age 45 are conditionally recommended for vaccination. Studies have shown that in transplant recipients whom would also be eligible and strongly considered to receive the HPV vaccine, only 32% of patients have received the HPV vaccine.^27^ Currently the Center of Disease Control (CDC) has published data exploring HPV vaccination uptake in the general population, in which 39% (including children) have received the HPV vaccine. In young adults, adults, this number remains low and only 47% of young adults have received at least one dose of the vaccine. In our study, we found that within patients aged 21–26, 59% had self-reported receiving the HPV vaccine. This may be a reflection of providers having a greater awareness about the importance of discussing HPV vaccination with patients, though other important differences would also exist between women living with SLE and the general population (including demographics). There were no differences in this study according to race/ethnicity.^28^ Our study is unique in that we identified certain factors (including the attitudes, knowledge and beliefs of patients using the HBM framework) that are associated with decreased vaccination which include age, Spanish-speaking patients, and perceived barriers, which has not been seen in previous studies exploring HPV vaccination in patients living with SLE. Of note, after adjusting for language in our multivariable regression model it had become non-significant – though this needs to be interpreted cautiously due to the small sample size of Spanish-speaking patients that received the HPV vaccine. However, this model did show that older patients had significantly reduced odds of receiving the HPV vaccine, reflecting that clinical care members should be cognizant of and offer HPV vaccination when appropriate. The totality of the current evidence suggest that HPV vaccine uptake is low in patients with SLE, and further strategies are needed to improve these rates.

To further determine why HPV vaccination rates are low in our patient population, we administered a knowledge and HBM questionnaire. Although patients were generally knowledgeable about HPV causing cervical cancer, patients were not generally aware that HPV can cause other symptoms (such as warts) and risk factors for HPV (24% and 26% respectively). Our knowledge questionnaire reaffirms the importance of educating patients during clinic appointments (by both primary care providers and rheumatologists) about HPV, cervical cancer, and the importance of SLE as a risk factor. Further research is needed to determine the best strategy to improve the education in patients living with SLE.

Our HBM questionnaire showed that *perceived barriers* including knowledge about how to receive the HPV vaccine, in-efficacy of the vaccine, and possible side effects were all correlated with decreased vaccination. Increased *Cues to Action* was also with increased vaccination as well. The HBM questionnaire highlights important strategies that can be used to improve vaccinations in this patient population such as offering the HPV vaccine in clinic, educating about potential adverse effects, and educating patients about the increased risk of HPV while living with SLE. Of note, these results are also similar to our previous study, that also demonstrates that *perceived barriers* in the HBM are significantly and moderately correlated with decreased cervical cancer screening.^13^

The strengths of this study include enrolling a racially and ethnically diverse population of women with SLE whom are predominantly on public insurance. Furthermore, despite low rates of HPV vaccination we had an adequate sample size to determine significant correlations with decreased vaccination, which can be used as strategies in the future to improve HPV vaccination rates. Limitations include that this was a cross-sectional study and that we cannot establish prospectively that our HBM model is associated with decreased vaccinations. Furthermore, HPV vaccination status was self-reported and not independently confirmed in the medical record, which introduce inherent biases. We also did not include male patients living with SLE in this study, and further research is needed to determine the vaccination rates within this patient population and whether there are different associations in the HBM between screening and non-screening. This is particularly important, as young male adults in the general population are significantly less likely to be vaccinated than females.^29^ Finally, the knowledge questionnaire that was developed is novel, and future studies will be needed to confirm whether there are gaps of knowledge pertaining to cervical cancer screening and HPV vaccination in women with SLE.

In conclusion, this study shows that women with SLE have low HPV vaccination rates, despite being at an increased risk for cervical cancer. Our HBM model and knowledge questionnaire show potential strategies that can improve HPV vaccination rates in this patient population. Finally, further research within this area is also needed to determine whether disease activity or medications may affect vaccine efficacy.

## References

[1] Dreyer L Faurschou M Mogensen M , et al. High incidence of potentially virus-induced malignancies in systemic lupus erythematosus: a long-term followup study in a Danish cohort. Arthritis Rheum 2011; 63: 3032–3037. 21953088 10.1002/art.30483

[2] Mendoza-Pinto C García-Carrasco M Vallejo-Ruiz V , et al. Incidence of cervical human papillomavirus infection in systemic lupus erythematosus women. Lupus 2017; 26: 944–951. 28059024 10.1177/0961203316686708

[3] Wadstrom H Arkema EV Sjowall C , et al. Cervical neoplasia in systemic lupus erythematosus: a nationwide study. Rheumatology 2017; 56: 613–619. 28039412 10.1093/rheumatology/kew459 PMC5850736

[4] García-Carrasco M Mendoza-Pinto C Rojas-Villarraga A , et al. Prevalence of cervical HPV infection in women with systemic lupus erythematosus: a systematic review and meta-analysis. Autoimmun Rev 2019; 18: 184–191. 30572140 10.1016/j.autrev.2018.09.001

[5] Moreno-Torres V Martínez-Urbistondo M Vázquez-Comendador J , et al. Higher mortality risk from gynaecological neoplasms and non-hodgkin's lymphoma in patients with systemic lupus erythematosus: an observational study from the Spanish national registry. Lupus Sci Med 2024; 11(1): e001153. 38631847 10.1136/lupus-2024-001153 PMC11029302

[6] Herrero R González P Markowitz LE . Present status of human papillomavirus vaccine development and implementation. Lancet Oncol 2015; 16: e206–216. 25943065 10.1016/S1470-2045(14)70481-4

[7] Infante V Miyaji KT Soarez PC , et al. Systematic review and meta-analysis of HPV vaccination in women with systemic lupus erythematosus (SLE). Expert Rev Vaccines 2021; 20: 309–318. 33573404 10.1080/14760584.2021.1889375

[8] Meites ESP Chesson HW Unger ER , et al. MMWR Morb Mortal Wkly Rep 2019.

[9] Murakawa Y Dobashi H Kondo M , et al. Questionnaire survey on the prevention and development of cervical cancer in patients with systemic lupus erythematosus in Japan. Mod Rheumatol 2023; 34(2): 352–358. 10.1093/mr/road028 36929382

[10] Dhar JP Walline H Mor G , et al. Cervical health in systemic lupus erythematosus. Womens Health Rep (New Rochelle) 2023; 4: 328–337. 37476603 10.1089/whr.2023.0023 PMC10354720

[11] Dhar JP Essenmacher L Dhar R , et al. Lack of uptake of prophylactic human papilloma virus vaccine among women with systemic lupus erythematosus seen at a regional medical center. J Clin Rheumatol 2019; 25: 348–350. 31764496 10.1097/RHU.0000000000000866

[12] Champion VL . Instrument development for health belief model constructs. ANS Adv Nurs Sci 1984; 6: 73–85. 6426380 10.1097/00012272-198404000-00011

[13] Bruera S Bowman S Huang Y , et al. Factors associated with adherence of cervical cancer screening in women with systemic lupus erythematosus. Arthritis Care Res 2024; 76: 1224–1231. 10.1002/acr.25355 38682616

[14] Patient Eligibility for Gold Card . Harris health system. Accessed 20 June 2023 at. https://www.harrishealth.org/access-care/patient-eligibility

[15] Aringer M Costenbader K Daikh D , et al. 2019 european league against rheumatism/american college of rheumatology classification criteria for systemic lupus erythematosus. Arthritis Rheumatol 2019; 71: 1400–1412. 31385462 10.1002/art.40930 PMC6827566

[16] Process of translation and adaptation of instruments 2016. https://www.who.int/substance_abuse/research_tools/translation/en/

[17] Meites E Szilagyi PG Chesson HW , et al. Human papillomavirus vaccination for adults: updated recommendations of the advisory committee on immunization practices. MMWR Morb Mortal Wkly Rep 2019; 68: 698–702. 31415491 10.15585/mmwr.mm6832a3 PMC6818701

[18] Jones CJ Smith H Llewellyn C . Evaluating the effectiveness of health belief model interventions in improving adherence: a systematic review. Health Psychol Rev 2014; 8: 253–269. 25053213 10.1080/17437199.2013.802623

[19] Schmotzer GL Reding KW . Knowledge and beliefs regarding human papillomavirus among college nursing students at a minority-serving institution. J Community Health 2013; 38: 1106–1114. 23813323 10.1007/s10900-013-9720-y PMC5501286

[20] Cheung T Lau JTF Wang JZ , et al. Acceptability of HPV vaccines and associations with perceptions related to HPV and HPV vaccines among male baccalaureate students in Hong Kong. PLoS One 2018; 13: e0198615. 29912883 10.1371/journal.pone.0198615 PMC6005511

[21] Guvenc G Akyuz A Acikel CH . Health belief model scale for cervical cancer and pap smear test: psychometric testing. J Adv Nurs 2011; 67: 428–437. 20946564 10.1111/j.1365-2648.2010.05450.x

[22] Hou SI Luh WM . Psychometric properties of the cervical smear belief inventory for Chinese women. Int J Behav Med 2005; 12: 180–191. 16083321 10.1207/s15327558ijbm1203_7

[23] Lau JT Wang Z Kim JH , et al. Acceptability of HPV vaccines and associations with perceptions related to HPV and HPV vaccines among men who have sex with men in Hong Kong. PLoS One 2013; 8: e57204. 23451188 10.1371/journal.pone.0057204 PMC3579800

[24] Yakout M Cervical S . Cancer and screening test (PAP test): knowledge and beliefs of Egyptian women. Am J Nurs Sci 2016; 5(5): 175.

[25] Montgomery K Smith-Glasgow ME . Human papillomavirus and cervical cancer knowledge, health beliefs, and preventive practices in 2 age cohorts: a comparison study. Gend Med 2012; 9: S55–66. 22340641 10.1016/j.genm.2011.11.002

[26] Bowden SJ Doulgeraki T Bouras E , et al. Risk factors for human papillomavirus infection, cervical intraepithelial neoplasia and cervical cancer: an umbrella review and follow-up Mendelian randomisation studies. BMC Med 2023; 21: 274. 37501128 10.1186/s12916-023-02965-w PMC10375747

[27] Vigil K Bhatnagar S Bell C , et al. Acceptance of HPV vaccination in kidney transplant recipients. OBM Transplantation 2018; 02: 017.

[28] Villarroel MA Galinsky AM Lu PJ , et al. Human papillomavirus vaccination coverage in children ages 9–17 years: United States, 2022. NCHS Data Brief, 2024, pp. 1–8. 38358336

[29] Chen MM Mott N Clark SJ , et al. HPV vaccination among young adults in the US. JAMA 2021; 325: 1673–1674. 33904878 10.1001/jama.2021.0725 PMC8080227

